# Parent, Partner, Co-Parent or Partnership? The Need for Clarity as Family Systems Thinking Takes Hold in the Quest to Motivate Behavioural Change

**DOI:** 10.3390/children4040029

**Published:** 2017-04-21

**Authors:** Chris May, Li Kheng Chai, Tracy Burrows

**Affiliations:** 1 School of Health Sciences, Faculty of Health, University of Newcastle, Callaghan, NSW 2308, Australia; LiKheng.Chai@uon.edu.au; 2 Priority Research Centre in Physical Activity and Nutrition, School of Health Sciences, Faculty of Health and Medicine, University of Newcastle, Callaghan, NSW 2308, Australia; tracy.burrows@newcastle.edu.au

**Keywords:** parenting, obesity, fathering, co-parenting, family-Systems, childhood, child, overweight

## Abstract

Research is increasingly pointing to the importance of extending the focus of childhood obesity intervention to include fathers, fathering figures, and other members of a child’s primary parenting network. Advances in communication technology are now making it possible to achieve this aim, within current resources, using modalities such text messaging, web-based resources and apps that extend intervention to parents not in attendance at face to face interactions. However, published research is often unclear as to which parent/s they targeted or engaged with, whether interventions planned to influence behaviours and capabilities across family systems, and how this can be achieved. As childhood obesity research employing information technology to engage with family systems takes hold it is becoming important for researchers clearly describe who they engage with, what they hope to achieve with them, and the pathways of influence that they aim to activate. This paper integrates extant knowledge on family systems thinking, parenting efficacy, co-parenting, and family intervention with the way parents are represented and reported in childhood obesity research. The paper concludes with recommendations on terminology that can be used to describe parents and parenting figures in future studies.

## 1. Introduction

Multiple studies have demonstrated that parents’ knowledge, attitudes, and behaviour influence children’s health and weight [1,2]. It is also evident that parental characteristics, such as gender, smoking, alcohol intake, body mass index, level of education, and other indicators of socio-economic status are linked to the risk of childhood obesity [2]. Furthermore, the influence that parents have on child outcomes is also determined by different parenting roles, the relationships that parents have with their children, and the way that parents work together in raising children. These factors may be particularly important for pre-adolescent children whose parents collectively determine what is eaten, how food is prepared, and where and when it is consumed [1,3,4]. However, published research is often unclear as to which parent/s they targeted or engaged with, whether interventions planned to influence behaviours and capabilities across family systems, and how this can be achieved [5,6,7,8,9,10,11]. This paper aims to (i) integrate extant knowledge on family systems thinking, parenting efficacy, co-parenting, and family intervention with the way parents are represented and reported in childhood obesity research; and (ii) provide recommendations on how parents and their relationships can be best described in future studies.

Families are complex interactive systems in which many players, across an array of contexts, exercise differing levels of influence on child outcomes. Bronfenbrenner was the first to describe an ecological perspective on family systems in which social and cultural layers exercise different levels of influence on children’s social, emotional, and intellectual development [12]. Bronfenbrenner predicted that the family’s microsystem, made up of children, parents, and other immediate carers, would exercise a major influence on child outcomes. Influence on child outcomes was predicted to decrease as other layers of influence: the mesosystem, including peers, teachers, sporting groups; the exosystem, including work, media, services and infrastructure; and the macrosystem of culture, subculture, and political systems, were predicted to become larger and more removed from the child’s care [12]. Therefore, it is important for researchers to clearly articulate and describe who they engage with, what they hope to achieve with them, and the pathways of influence that they aim to activate.

## 2. Research on Childhood Obesity Research Skewed towards Maternal Participation

Studies designed to influence children’s eating behaviours often describe engagement with “parents” but this can be misleading when parent participation is heavily skewed toward mothers. A recent systematic review seeking to assess father involvement in paediatric obesity prevention trials found that explicit attempts to engage with fathers occurred in just <1% of eligible trials (*N* = 213). Morgan et al. also found that fathers accounted for only 6% of parents in studies limited to single-parent participation (*n* = 133) and that only 2% of eligible papers identified a lack of paternal participation as a potential limitation [13]. Systematic reviews of both quantitative and qualitative research on family-focused childhood obesity studies (*N* = 667 studies) found that 51% of these studies included both mothers and fathers, 37% included mothers only, and 1% included fathers only [10,14]. Although slightly more than half of these studies included fathers with/without mothers, only 10% reported maternal and paternal results separately [14,15,16]. In studies reporting to engage with both parents, the mean sample size for mothers (*n* = 672) was almost five times greater than fathers (*n* = 139) [14]. It is, therefore, reasonable to assume that the remaining studies (11%) that did not report on parent gender were also heavily skewed toward maternal data [10,14]. Reports can be easily misinterpreted when cohorts are described as parents, but the conclusions are primarily founded on maternal data. More accurate description of parent participants will be required as research in childhood obesity and family studies increasingly explores the importance of parenting roles and relationships in determining child outcomes.

## 3. Family Systems and Behaviour Change

Parenting efficacy forms the cornerstone of most of the studies that engage with parents in the hope of achieving change in weight-related behaviours. Parenting self-efficacy (PSE) describes a parent’s belief in their own ability to perform well in the parenting role [17,18]. A parent’s sense of PSE plays an important role in motivating them to do their best in helping their children to achieve optimal developmental outcomes [19,20,21]. Interventions targeted at reducing childhood obesity often aim to influence PSE by enhancing parent knowledge about factors, such as food selection, preparation, presentation, and eating behaviours [22]. However, research in family studies has found that knowledge is a relatively weak predictor of PSE when compared to other factors, such as parenting stress, general self-efficacy, partner support, and other measures of family function [18]. It is, therefore, important for researchers and practitioners to take these and other factors, such as parent gender, into account and describe how their planned intervention is intended to influence parenting efficacy in the context of the family system.

Societal changes have created expectations that the roles and responsibilities that parents assume will not be determined by gender, yet evidence pointing to the persistence of gendered parenting roles and responsibilities is overwhelming [23,24]. Setting functional determinants, such as the ability to breastfeed, aside, the available evidence suggests that there is little difference between mothers and fathers when it comes to their ability to either nurture their children, or care for their physical needs [25]. However, longstanding factors that influence the division of roles and responsibilities in families continue to predetermine, encourage, and reinforce paradigms of gendered parenting responsibility [26]. The most obvious of these are parents’ commitments to paid work, a factor that directly affects the time that mothers and fathers interact in dyadic relationships with their children.

**Figure 1:**
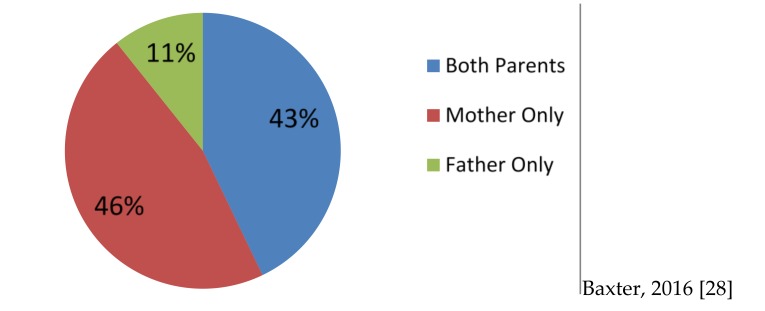
Parents’ share of the time they spend with their children in two-parent families.

Australian mothers, like those in other high income countries, have taken on more paid work over the last half century [23]. This has had a positive influence on both the time that fathers spend with their children and the expectations that fathers have for involvement in their parenting role. However, the gendered influence of paid work on family dynamics remains substantial [23,27]. A recent analysis of data from the Longitudinal Study of Australian Children (unpublished data presented at Fathering Symposium 2016 by Baxter) [28] illustrates how strongly gendered roles and responsibilities continue to influence the amount of time that mothers and fathers spend alone with their children in two parent households. The analysis (Figure 1) shows that children spent almost 50% of their time with parents alone with their mother, that mothers were present for almost all the time that children were with their parents (89%), and that fathers could expect to experience dyadic 1:1 involvement with their children for 11% of this time. This analysis demonstrates that children in these families spent much of their time with parents in the rich triadic relationship that they share with both parents and that fathers can often expect to perform most of their direct parenting work in this triadic context.

## 4. The Importance of Parenting Partnerships

**Figure 2:**
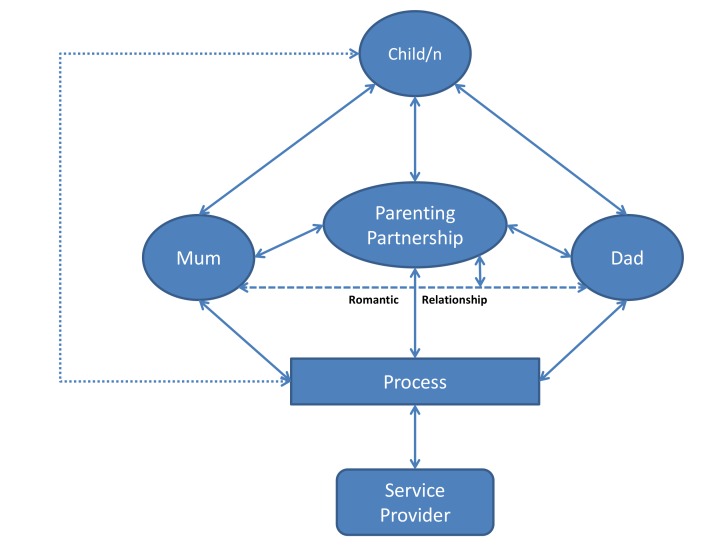
An interactive effects model of intervention in family services.

Although children have consequential dyadic relationships with each of their parents, they also develop important triadic relationships with their parents’ partnership [29,30]. Theorists and family researchers in a wide range of cultures have adopted the term “co-parenting” to describe the unique relationship that operates between parents as they work together in raising children [31,32,33]. While there is a degree of interplay between the co-parenting relationship and parents’ romantic or other relationships, the co-parenting relationship is an independent, measurable construct which can be strong and supportive when other aspects of a relationship are less successful [34,35]. The co-parenting relationship, therefore, forms an alternative entity within the family system and children have different relationships with this entity than they do with either of their parents. The quality of co-parenting relationships (otherwise referred to as parenting partnerships) has an independent influence on children’s social and emotional development (Figure 2), and factors that are likely to influence children’s risks of being overweight, but a recent review found no reports on interventions focused on co-parenting and childhood obesity [13].

Satisfaction with the support that parents receive from their parenting partner is a key determinant of how well this relationship works for both parents and children. Feinberg captured the importance of partner support when describing the co-parenting relationship as the “support and coordination (or lack of it) that parental Figures exhibit in childrearing” (p. 96) [31]. However, Feinberg also identified other factors that work together to represent the quality of co-parenting relationships: joint family management, support/undermining, childrearing agreement, and the distribution of parenting roles and responsibilities [31]. Although co-parenting theorists report minor disagreement about factors that make up their multivariate co-parenting models, there is also substantial similarity [33,36]. A general acceptance of these relatively discrete components of co-parenting has enabled the development of tools for assessing the quality of co-parenting relationships and, therefore, measuring relations between co-parenting quality and other factors [32,33,34]. The maturation of co-parenting theory has, thereby, supported the development of an increasingly complex body of evidence regarding associations that exist between co-parenting quality and both parent and child outcomes [37,38,39].

## 5. Parenting Partnerships and PSE

The potential importance of the co-parenting relationship in childhood obesity intervention is best explained by relations that have been found between partner support and PSE. A 2005 review of 47 studies exploring determinants of PSE concluded that support from significant others had a stronger association with PSE than parenting knowledge or any other factor [40]. Studies have shown that the primary source of support for most parents comes from the relationship they share with their parenting partner [41,42,43]. Therefore, the strength of maternal or paternal PSE is best predicted by the availability and quality relationships that support parents in their parenting roles. Thus, child obesity interventions that focus on enhancing a parent’s knowledge and skills, but overlook the importance of engaging both parents in behaviour change, are unlikely to have a substantial influence on parents’ beliefs that they will be able to achieve meaningful change in their child’s eating behaviours. 

## 6. Partnership-Inclusive Practice

Evidence about the importance of parenting partnerships in determining child and parent outcomes has been used to call for practitioners to find ways to better engage and work with parenting partners—usually fathers. However, despite a range of possible pathways for interaction between services and families, as illustrated in Figure 2, it has proven to be difficult to shift practice toward greater direct engagement with fathers or parenting partnerships. A major reason for this difficulty is thought to be the way that the processes and practices that support the provision of service in the family sector have developed with ingrained expectations of close working relationships between practitioners and mothers [26]. Recommendations for father inclusion usually focus on barriers, such as the timing of service delivery and the feminisation of services. However, tinkering with these factors is unlikely to have a substantial influence on father inclusion because of persistent gender-related expectations held by practitioners, parents, and the broader community [9,26]. For example, it has been shown that employers tend to expect mothers to request and take leave to attend to family matters, and see this as a sign of appropriate nurturing behaviour, but discourage fathers from attending such events and view their requests to do so as a sign of poor work commitment [23]. These and other barriers to partner participation are deeply rooted in present-day culture to the point where similar attendance patterns also occur in community-based programs [9]. It is, therefore, incumbent for service providers, who wish to engage with parenting partners and their partnerships, to find alternative and complementary ways of doing so.

Developments in childhood obesity intervention are increasingly turning toward family-focused interventions while struggling with the difficulty of engaging with parenting partners and parenting partnerships. For example, Davidson et al. reported reduced weight, increased physical activity, and reduced screen time in children (*N* = 423) whose parents participated in a pilot study linked to the Head Start Program and designed to focus on family systems [44]. Parents in this study were engaged in a participatory research design process to enhance their engagement, however, the study engaged with 88 mothers, six grandmothers, and only six fathers. Ward et al. described an intervention designed to integrate a family focused approach into childhood obesity prevention but the program based design of this intervention is unlikely to result in significant recruitment and retention of fathers [45]. Healthy Dads, Healthy Kids (HDHK) has reported that a program focused on fathers’ health and well-being in the context of his relationship with his child can have a positive influence on both child and father weight and eating behaviours [46]. These and other studies have made important contributions to an understanding of how to work with families in addressing childhood obesity, but these programs are labour-intensive, require highly-specialised skills and have not demonstrated that they can effectively engage with all members of the family microsystem.

Information technology (IT), such as mobile phones and digital communication are proving to be a potential way forward in engaging with family systems in research and service delivery [47]. Recent evidence indicates that fathers and mothers are likely to engage, and remain engaged, with messaging systems that provide family focused information, encouragement, support, and links to further resources [48,49]. However, more research is required to determine if these or similar systems can have an influence on personal or collective parenting efficacy. Interventions relying on these technologies are highly scalable and able to function alongside current systems without diverting substantial resources or requiring organisational change.

Messaging systems can also be adapted to engage parenting partners in a variety of contexts. Although the majority of parenting partnerships occur between biological (mother and father) couples, parenting partnerships also occur in same-sex relationships, between parents and grandparents, and in a range of other contexts. Systems founded in IT can be oriented to respond to information about participants and nuance messaging to fit with stages of family development and the contexts in which different parenting partnerships operate. As scalable and adaptable IT-based interventions develop, along with the technologies that support them, it can be expected that research will increasingly focus on interventions designed to engage with fathers and other members of the family system. It will, therefore, become increasingly important for researchers to accurately describe who the participants are and how interventions are expected to influence behavioural change.

## 7. Recommendations for the Terminology Used to Describe Parents

**Figure 3:**
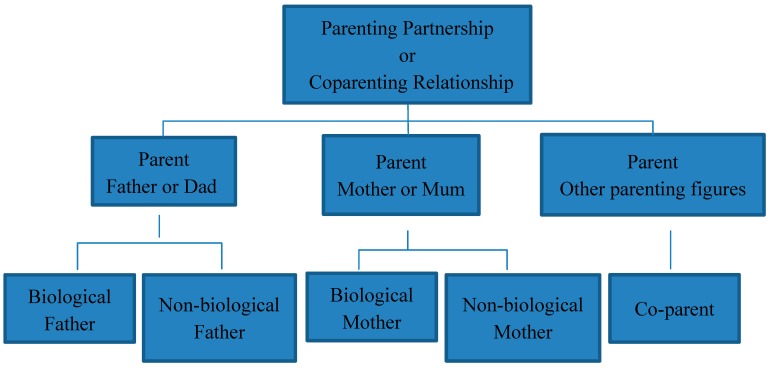
A proposed taxonomy of parent terminology.

The available literature provides a reasonable taxonomy for describing a range of parenting participants (see Figure 3). While the term “parents” is a catch-all for any member of parenting partnerships, interventions that work with either mothers or fathers should describe participants using these terms, and analyse their data accordingly, because mothers and fathers make unique contributions to child outcomes [50]. Mothers and fathers can further be subdivided as biological and non-biological parents because this aspect of their relationship influences the way that parents interact with each other and with their children [51]. The term co-parent has been used to describe all parents, has often been used to describe divorced and separated parents, and is increasingly being used, in the formal and informal literature, to describe parenting partners who are not the child’s biological parent [52]. The present taxonomy aims to resolve some of the potential ambiguity that occurs when describing parents by providing a framework that fits with extant usage and supports researchers in clearly identifying gendered parenting roles and biological relationships. The use of terminology that accurately describes participating parents will have important implications as studies and subsequent reviews aim to understand the influence that interventions have on child and family outcomes.

This taxonomy does not address how the term co-parenting, as opposed to co-parent, should be used when describing participants. Interventions with a co-parent or parenting partner should only be described as a co-parenting intervention when they focus on the relationship that parenting partners share in the raising of children. For example, an intervention designed to give one or both parents information about vaccination cannot reasonably be described as a co-parenting intervention unless it aims to influence the way that parents work together in relation to vaccination. Although the term co-parenting is not usually used to describe the role of grandparents, aunts, uncles, and others who perform parenting roles, it may be reasonable to use co-parenting in these and other contexts if the focus of intervention is on the relationships that people share with parents in the raising of children [53].

## 8. Conclusions

Studies aiming to influence children’s weight-related behaviours are more likely to be successful when interventions enhance parenting self-efficacy or co-parenting competence in the procurement, preparation, and presentation of a healthy diet. Although available evidence points to the importance of interventions that take a family systems approach in research design, it is evident that the majority of studies continue to focus on mothers and fail to describe their intended mechanisms of influence. As technology enables practitioners to move away from traditional relationships with mothers and engage effectively with fathers and other family members, it is becoming more important for researchers to clearly describe the family members that they aim to engage with. The terminology that researchers use to describe members of the family system when researching childhood obesity and other nutrition disorders can be supported by an expanding literature on the importance of gender, parenting partnerships, and the range of contexts in which parenting occurs.

## References

[1] Van Der Kruk J.J., Kortekaas F., Lucas C., Jager-Wittenaar H. (2013). Obesity: A systematic review on parental involvement in long-term European childhood weight control interventions with a nutritional focus. Obes. Rev..

[2] Walsh B., Cullinan J. (2015). Decomposing socioeconomic inequalities in childhood obesity: Evidence from Ireland. Econ. Hum. Biol..

[3] Epstein L.H., Paluch R.A., Roemmich J.N., Beecher M.D. (2007). Family-Based Obesity Treatment, Then and Now: Twenty-Five Years of Pediatric Obesity Treatment. Health psychology: Official journal of the Division of Health Psychology, American Psychological Association. Health Psychol..

[4] Oude Luttikhuis H., Baur L., Jansen H., Shrewsbury V.A., O’Malley C., Stolk R.P., Summerbell C.D. (2009). Interventions for treating obesity in children. Cochrane Database Syst. Rev..

[5] Loveman E., Al-Khudairy L., Johnson R.E., Robertson W., Colquitt J.L., Mead E.L., Rees K. (2015). Parent-only interventions for childhood overweight or obesity in children aged 5 to 11 years. Cochrane Database Syst. Rev..

[6] Ewald H., Kirby J., Rees K., Robertson W. (2014). Parent-only interventions in the treatment of childhood obesity: A systematic review of randomized controlled trials. J. Public Health.

[7] Jang M., Chao A., Whittemore R. (2015). Evaluating Intervention Programs Targeting Parents to Manage Childhood Overweight and Obesity: A Systematic Review Using the RE-AIM Framework. J. Pediatr. Nurs..

[8] Berlyn C., Wise S., Soriano G. (2008). Engaging Fathers in Child and Family Services: Participation, perceptions and good practice. Fam. Matters.

[9] Fletcher R., May C., St George J., Stoker L., Oshan M. (2014). Engaging Fathers: Evidence Review.

[10] Gicevic S., Aftosmes-Tobio A., Manganello J.A., Ganter C., Simon C.L., Newlan S., Davison K.K. (2016). Parenting and childhood obesity research: A quantitative content analysis of published research 2009–2015. Obes. Rev..

[11] McAllister F., Burgess A., Kato J., Barker G. (2012). Fatherhood: Parenting Programmes and Policy—A Critical Review of Best Practice.

[12] Bronfenbrenner U. (1986). Ecology of the Family as a Context for Human Development: Research Perspectives. Dev. Psychol..

[13] Morgan P.J., Young M.D., Lloyd A.B., Wang M.L., Eather N., Miller A., Pagoto S.L. (2017). Involvement of Fathers in Pediatric Obesity Treatment and Prevention Trials: A Systematic Review. Pediatrics.

[14] Davison K.K., Gicevic S., Aftosmes-Tobio A., Ganter C., Simon C.L., Newlan S., Manganello J.A. (2016). Fathers’ Representation in Observational Studies on Parenting and Childhood Obesity: A Systematic Review and Content Analysis. Am. J. Public Health.

[15] Freeman E., Fletcher R., Collins C.E., Morgan P.J., Burrows T., Callister R. (2012). Preventing and treating childhood obesity: Time to target fathers. Int. J. Obes..

[16] Morgan P.J., Lubans D.R., Callister R., Okely A.D., Burrows T.L., Fletcher R., Collins C.E. (2011). The ‘Healthy Dads, Healthy Kids’ randomized controlled trial: Efficacy of a healthy lifestyle program for overweight fathers and their children. Int. J. Obes..

[17] Coleman P.K., Karraker K.H. (2000). Parenting Self-Efficacy Among Mothers of School-Age Children: Conceptualization, Measurement, and Correlates*. Fam. Rel..

[18] Sevigny P.R., Loutzenhiser L. (2010). Predictors of parenting self-efficacy in mothers and fathers of toddlers. Child Care Health Dev..

[19] Spielman, V., Taubman-Ben-Ari, O. (2009). Parental self-efficacy and stress-related growth in the transition to parenthood: A comparison between parents of pre- and full-term babies. Health Soc. Work.

[20] Bandura A. (1997). Self Efficacy: The Exercise of Control.

[21] Duncanson K., Burrows T., Collins C. (2013). Effect of a low-intensity parent-focused nutrition intervention on dietary intake of 2- to 5-year olds. J. Pediatr. Gastroenterol. Nutr..

[22] Collins C.E., Okely A.D., Morgan P.J., Jones R.A., Burrows T.L., Cliff D.P., Baur L.A. (2011). Parent diet modification, child activity, or both in obese children: An RCT. Pediatrics.

[23] Crabb A. (2015). The Wife Drought.

[24] Duncanson K., Burrows T., Holman B., Collins C. (2013). Parents’ perceptions of child feeding: A qualitative study based on the theory of planned behavior. J. Dev. Behav. Pediatr..

[25] Spinney L. (2011). Venus and Mars collide. New Sci..

[26] Frascarolo F., Feinberg M., Albert Sznitman G., Favez N. (2016). Professional gatekeeping toward fathers: A powerful influence on family and child development. http://www.waimh.org/files/Perspectives%20in%20IMH/2016_3/4-7_Frascaroloetal_2016_3_Perspectives_IMH.pdf7_Frascaroloetal_2016_3_Perspectives_IMH.pdf.

[27] Sayer L.C., Bianchi S.M., Robinson J. (2004). Are Parents Investing Less in Children? Trends in Mothers’ and Fathers’ Time with Children. Am. J. Sociol..

[28] Baxter A. Australian Institute of Family Studies. Presented at Fathering Research Symposium, University of Newcastle.

[29] Belsky J. (1984). The Determinants of Parenting: A Process Model. Child Dev..

[30] Flouri E., Buchanan A. (2004). Early father’s and mother’s involvement and child’s later educational outcomes. Br. J. Educ. Psychol..

[31] Feinberg M.E. (2003). The Internal Structure and Ecological Context of Co-parenting: A Framework for Research and Intervention. Parent. Sci. Pract..

[32] McHale J.P., Kuersten-Hogan R. (2004). Introduction: The Dynamics of Raising Children Together. J. Adult Dev..

[33] Van Egeren L.A., Hawkins D.P. (2004). Coming to Terms with Co-parenting: Implications of Definition and Measurement. J. Adult Dev..

[34] Feinberg M.E., Brown L.D., Kan M.L. (2012). A Multi-Domain Self-Report Measure of Co-parenting. Parent. Sci. Pract..

[35] Morrill M.I., Hines D.A., Mahmood S., Cordova J.V. (2010). Pathways between marriage and parenting for wives and husbands: The role of co-parenting. Fam. Process.

[36] McHale J.P., Kuersten-Hogan R., Rao N. (2004). Growing Points for Co-parenting Theory and Research. J. Adult Dev..

[37] Feinberg M.E., Jones D.E., Kan M.L., Goslin M.C. (2010). Effects of family foundations on parents and children: 3.5 years after baseline. J. Fam. Psychol..

[38] Schoppe S.J., Mangelsdorf S.C., Frosch C.A. (2001). Co-parenting, family process, and family structure: Implications for preschoolers’ externalizing behavior problems. J. Fam. Psychol..

[39] Teubert D., Pinquart M. (2010). The association between co-parenting and child adjustment: A meta-analysis. Parent.: Sci. Pract..

[40] Jones T.L., Prinz R.J. (2005). Potential roles of parental self-efficacy in parent and child adjustment: A review. Clin. Psychol. Rev..

[41] Leerkes E.M., Burney R.V. (2007). The Development of Parenting Efficacy Among New Mothers and Fathers. Infancy.

[42] Yu M. Family Relationships Quarterly No. 19: Australian Institute of Family Studies. https://aifs.gov.au/cfca/publications/family-relationships-quarterly-no-19/parenting-efficacy-how-can-service-providers-help..

[43] May C.D. The importance of co-parenting quality when parenting a child with an Autism Spectrum Disorder: A mixed method investigation 2014. http://nova.newcastle.edu.au/vital/access/manager/Repository/uon:15001.

[44] Davison K.K., Jurkowski J.M., Li K., Kranz S., Lawson H.A. (2013). A childhood obesity intervention developed by families for families: Results from a pilot study. Int. J. Behav. Nutr. Phys. Act..

[45] Ward D.S., Vaughn A.E., Bangdiwala K.I., Campbell M., Jones D.J., Panter A.T., Stevens J. (2011). Integrating a family-focused approach into child obesity prevention: Rationale and design for the My Parenting SOS study randomized control trial. BMC Public Health..

[46] Morgan P.J., Collins C.E., Plotnikoff R.C., Callister R., Burrows T., Fletcher R., Cook A.T. (2014). The ‘Healthy Dads, Healthy Kids’ community randomized controlled trial: A community-based healthy lifestyle program for fathers and their children. Prev. Med..

[47] Eapen Z.J., Peterson E.D. (2015). Can Mobile Health Applications Facilitate Meaningful Behavior Change?. Time Answ. JAMA.

[48] May C., Fletcher R. The Development and Application of A Protocol for Writing, Assessing and Validating A Corpus of Relationship-Focused Text Messages for New and Expecting Fathers. Health Informatics.

[49] Whittaker R., Matoff-Stepp S., Meehan J., Kendrick J., Jordan E., Stange P., Ratzan S. (2012). Text4baby: Development and Implementation of a National Text Messaging Health Information Service. Am. J. Public Health.

[50] Cabrera N.J., Fitzgerald H.E., Bradley R.H., Roggman L. (2014). The Ecology of Father-Child Relationships: An Expanded Model. J. Fam. Theory Rev..

[51] Favez N., Widmer E.D., Doan M.-T., Tissot H. (2015). Co-parenting in Stepfamilies: Maternal Promotion of Family Cohesiveness with Partner and with Father. J. Child Fam. Stud..

[52] Merriam-Webster (2016). Dictionary: Co-parent [Internet]. https://www.merriam-webster.com/dictionary/co-parent..

[53] Secretariat of National Aboriginal and Islander Child Care Child Rearing Practices 2016. http://www.supportingcarers.snaicc.org.au/caring-for-kids/child-rearing-practices/.

